# Supplementary figures and images for: Genome-wide identification of the bZIP transcription factor family and expression analysis under abiotic stress in Zanthoxylum bungeanum
Source: PLoS One. 2025 May 29;20(5):e0324447. doi: 10.1371/journal.pone.0324447 (PMC12122043; doi:10.1371/journal.pone.0324447)

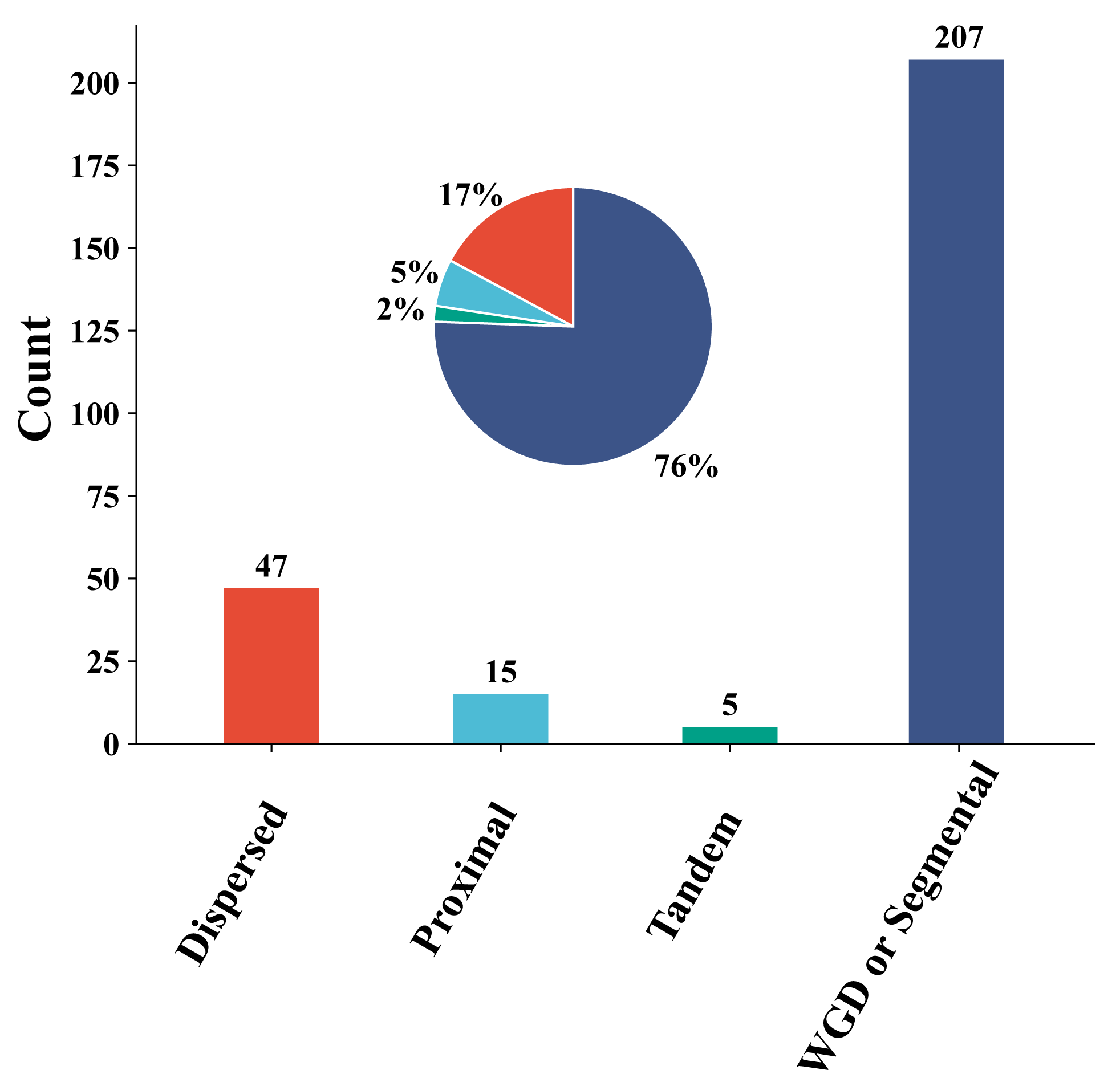

Supplement: S1 Fig — The same color in the pie chart and the histogram represents the same replication event. (TIFF) [file pone.0324447.s001.tif]

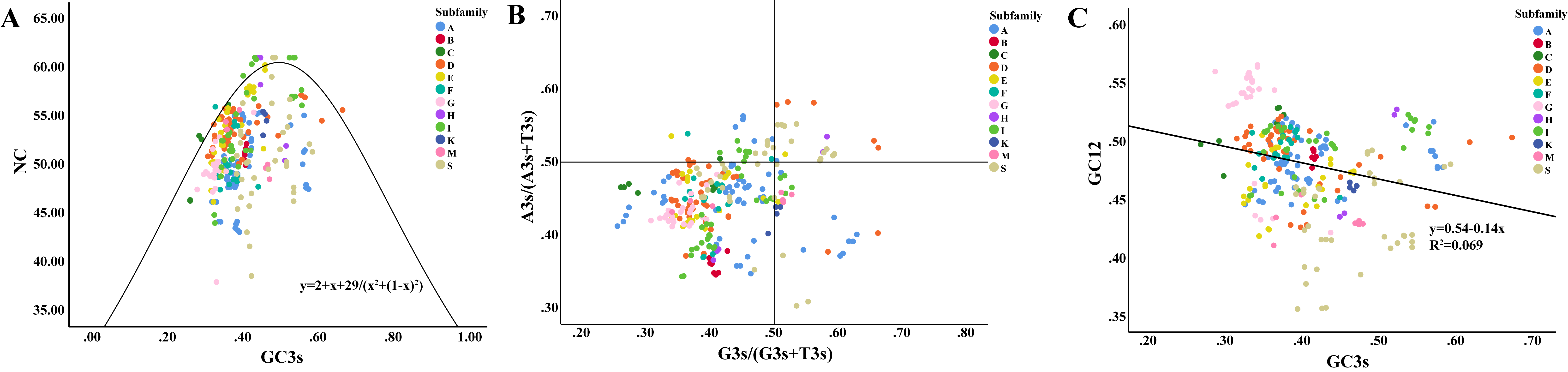

Supplement: S4 Fig — (A) ENC map analysis. (B) PR2 plot analysis. (C) Neutral plot analysis. (TIFF) [file pone.0324447.s004.tif]

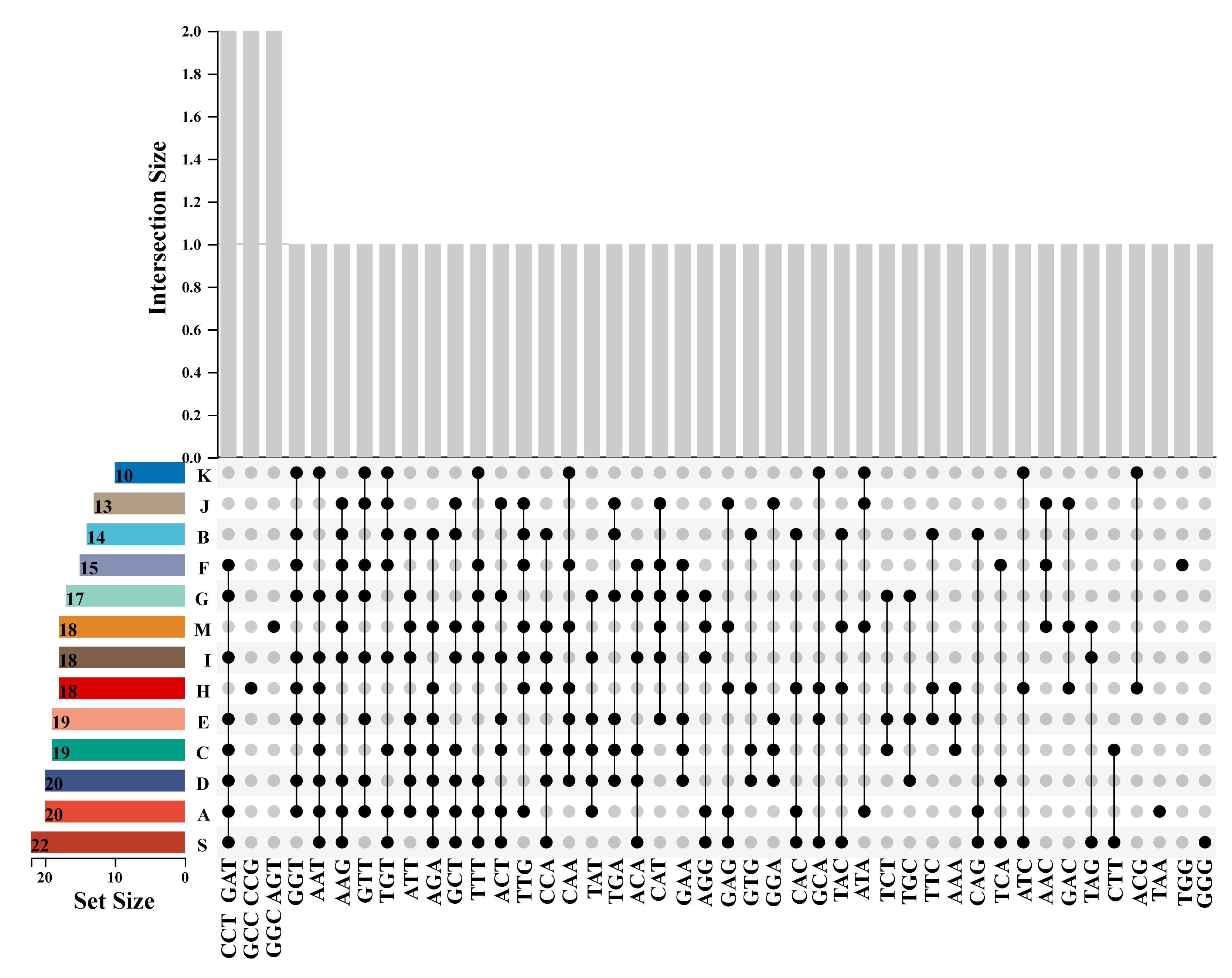

Supplement: S5 Fig — The bar chart (top) represents the number of italicized codons below. The black dots in the graph (bottom) indicate that the corresponding codon is the optimal codon of the corresponding subfamily. The measured color bands and numbers on the left represent the number of optimal codons in the corresponding subfamily. (TIFF) [file pone.0324447.s005.tif]

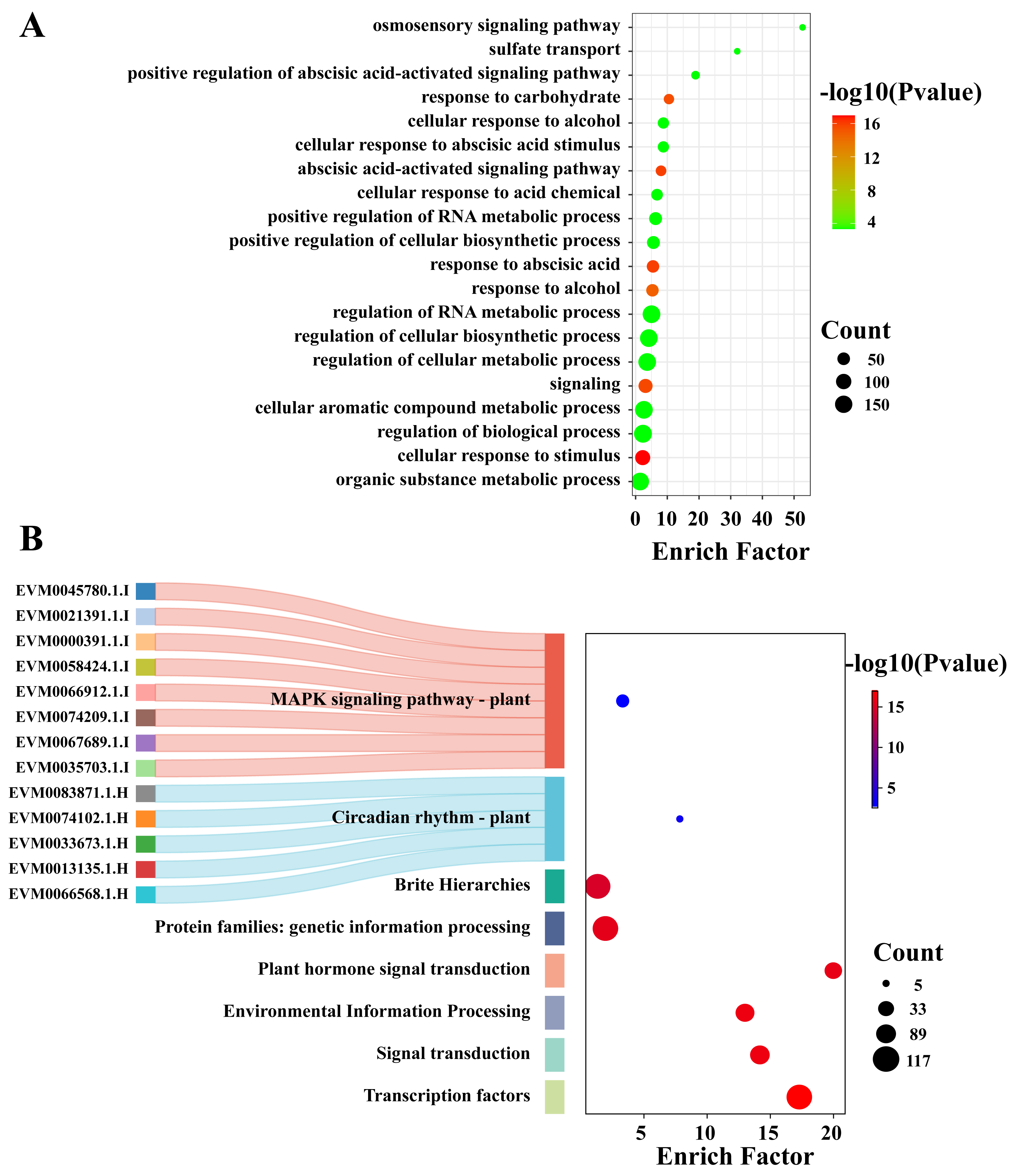

Supplement: S6 Fig — (A) GO enrichment analysis of ZbbZIPs. (B) KEGG enrichment analysis of ZbbZIPs. (TIFF) [file pone.0324447.s006.tif]
